# Leveraging genomic diversity to promote human and animal health

**DOI:** 10.1038/s42003-019-0708-8

**Published:** 2019-12-11

**Authors:** Michèle Ramsay, Han G. Brunner, Appolinaire Djikeng

**Affiliations:** 1 Sydney Brenner Institute for Molecular Bioscience, Faculty of Health Sciences, University of the Witwatersrand, Johannesburg, South Africa; 2 Department of Clinical Genetics and GROW-School for Oncology and Developmental Biology, Maastricht University Medical Center, Maastricht, The Netherlands; 3 Department of Human Genetics, Donders Institute for Brain, Cognition, and Behaviour, Radboud University Medical Center, Nijmegen, The Netherlands; 4 Centre for Tropical Livestock Genetics and Health, The University of Edinburgh, Scotland, UK

**Keywords:** Research data, Animal breeding

## Abstract

Genomic diversity is a driving force influencing human and animal health, and susceptibility to disease. During the Keystone Symposium on *Leveraging Genomic Diversity to Promote Human and Animal Health* held in Kampala on Lake Victoria in Uganda, we brought together diverse communities of geneticists with primary objectives to explore areas of common interest, joint technological and methodological developments and applications, and to leverage opportunities for cross-learning. We explored translational genomics research in farmed animals and humans, debated the differences in research objectives in high- and low-resourced environments, delved into infectious diseases and zoonoses affecting humans and animals and considered diversity and cultural context at many levels. The 109 participants were from 22 countries (13 in Africa) and included 44 global travel awardees from 9 countries, equal numbers of men and women, of whom 31 were students and 13 senior investigators.

## Meeting background

There are fundamental differences in the objectives of human genetics and animal genetics and breeding. Human genetics is focused on the individual and the need to make an accurate diagnosis that will inform the best treatment to ensure the best achievable health outcomes for the individual and their immediate family. It is about providing biological insights, developing more effective treatments and giving parental couples reproductive choices. On the other hand, animal genetics and breeding are focused on the performance of single or groups of animals against predetermined and accurately measurable parameters including but not limited to productivity, adaptation/resilience, growth, tolerance/susceptibility to diseases, feed conversion and energy utilization. This is achieved through the selection of desirable traits, a process that has developed over several millennia, since the domestication of animals, and the field has experienced dramatic acceleration over the past two decades with the advent of the genomics era, as explained by Michel Georges. There have been remarkable successes in animal breeding, leading to considerable genetic improvement and gains supporting productivity (e.g. milk, meat and egg production), adaptation, growth and other key traits. These significant genetic gains have also brought about an added environmental bonus in the form of a reduced carbon footprint. This report provides some of the highlights and insights from the deliberations during the four-day meeting.

## Common tools and approaches between human genetics and animal genetics and breeding

Since our understanding of genome–phenome correlation is premised on good data, there were debates about the most suitable technologies to provide us with accurate reference genomes to stimulate the discovery of novel insights and a more accurate understanding of the biological mechanisms. Long-range sequencing and single-molecule sequencing technologies are coming of age and promise to provide more accurate and complete reference genomes. This will ensure that the genomes of all species are more fully characterized, especially with regard to copy-number variants (CNVs) and other structural variations that are predicted to have a profound impact on biological processes.

Evolution and genome adaptation were a constant refrain throughout the sessions. Evan Eichler presented the Keynote Address on the relevance of primate evolution to human and animal health. He explained that segmental duplications, ranging upwards from 50 bp (with CNVs now having been redefined to a lower threshold from the previous 1000-bp limit) have been understudied in most species as they are often intractable when analyzing short-read sequences. The duplication events can occur within chromosomes (intra-segmental duplications) or between chromosomes (inter-segmental duplications) and create regions of potential instability and hotspots for non-homologous crossover events. All primates, including humans, have extensive intra- and inter-segmental duplication events, whereas all other animals that have been studied have abundant intra- but relatively fewer inter-segmental duplication events. Hitching a ride with these events are gene families that have expanded uniquely in humans and include genes involved in immune response and toxin exposure.

In humans, signals of selection reflect local and regional adaptations to diet (*AMY1*—amylase gene), to increased levels of arsenic in ground water supplies (*AS3MT*) and to pathogen infections (Lassa fever, Yellow fever, malaria, trypanosomiasis and dengue). Charles Rotimi explained that the protective alleles often reach high frequencies in regions of the highest and longest exposure and when people migrate out to other geographic locations, they take the variants with them. In admixed populations, specific variants can be correlated with the percentage of ancestry from a specific location. One example is where lower levels of LDL cholesterol and higher levels of triglycerides are correlated with an increased proportion of African ancestry. Louisa Pereira brought fascinating insights showing that an increased level of population-based genetic resistance to dengue was correlated with African ancestry.

## Integrating big data, machine learning and artificial intelligence to investigate and establish linkages between genotypes and phenotypes

Large public genomic and other omic resources so familiar in the human research landscape (e.g. 1000 Genomes Project, ENCODE, GTEx, the UK Biobank and many others), with portals for sharing the outcomes of massive analyses (e.g. providing information through genome browsers like https://www.ensembl.org/index.html and https://genome.ucsc.edu/, and http://geneatlas.roslin.ed.ac.uk/) are paralleled in the animal research community by, for example, the 1000 Bull Genomes Project (http://www.1000bullgenomes.com/), the International Goat Genome Consortium (http://www.goatgenome.org) and poultry genome consortia, as elaborated on by Niklas Blomberg. These initiatives pool data and resources to develop databases and tools for research, including SNP genotyping arrays for genome-wide association studies, and set standards for global data sharing (e.g. The Global Alliance for Genomics and Health (GA4GH)—https://www.ga4gh.org/). Both the animal and human genetics communities are well aware of the need for systems biology research approaches and are developing resources that explore epigenomes, transcriptomes, eQTLs and microbiomes in the context of health and disease, as well as agricultural productivity in the case of animals. International data resources are receiving much attention, and different models and processes used to assess data are being adopted. These vast data resources provide opportunities for machine-learning approaches, by integrating human, animal and pathogen data, and promote a better understanding of the linkages between genotypes and phenotypes. Some discussion points from the meeting on different approaches to single-gene disorders and complex multifactorial diseases are given below.

Understanding the genetic basis of monogenic traits is important for several reasons: providing an accurate diagnosis, guiding treatment, providing alternate therapeutic approaches and informing reproductive choices, as discussed by Han Brunner. The wide application of next-generation sequencing technologies has revealed that every baby is born with 70–100 novel mutations, not present in either parent. Although often benign, these mutations could lead to serious disorders. While recessive diseases dominate in populations with high consanguinity, new mutations are the main cause of neurodevelopmental disorders such as intellectual disability and autism in outbred populations. The links between genetic mutation and phenotype are increasingly being uncovered in the field of medical genetics and play an important role in patient management and care. Brunhilde Wirth used the example of spinal muscular atrophy to explain how recent advances and knowledge of the genetics of disease has helped to develop more targeted therapies, by presenting both significant challenges and much promise.

In the world of animal genetics and breeding, there are good examples of monogenic traits or large-effect variants that have been sought by breeders (e.g. polled cattle, hair coverage and length and PRRS resistance in pigs, as explained by Simon Lillico). Attempts to establish the desired alleles to fixation in herds have sometimes been hampered by the hitch-hiking of undesirable traits. Genome-editing technologies are now being considered as they target only the gene of interest, but this approach has led to safety and ethical concerns among sectors of the public as illustrated in the presentation by Alison van Eenennaam. It is, however, a potentially revolutionary technology for boosting agricultural production and lowering costs and adverse environmental impacts.

In the animal world, it is common to refer to breeding value and preservation of germplasm of individuals and their potential impact on the development of the herd. John Hickey led us through the process of how a deep understanding of the relationship between the genetic variants and the phenotype can be applied to the notion that an embryo’s potential value can be determined even before the phenotype is fully formed. Although there are important monogenic traits relevant to animal breeding, most desirable traits are polygenic and multifactorial, like milk production and fat content in cattle. The shorter generation time and control over the environment, for example in terms of diet, temperature and exposures, make it more tractable to assess and predict the phenotypic outcome among animals. David Evans provided excellent explanations on how to assess genetic causality in the context of complex traits. Susan Lamont spoke about genetic associations for traits in poultry breeding and Mark Fife on the diversity of immune loci in Europe and Africa.

On the other hand, in terms of public health applications for genomics, Michele Ramsay explained that there is much interest in polygenic risk scores (PRSs), with the purpose of stratifying human populations to identify those at the highest risk for specific diseases. This would provide opportunities for interventions to prevent or ameliorate disease through targeted treatment. The consensus at the meeting was that this is not yet a practical approach, especially for understudied populations like those in Africa. We were also reminded that PRSs can only be as good as the proportional heritability of the trait under investigation, a point often ignored or minimized by proponents of genomic medicine in the context of complex traits.

## Landscape genomics: is there a human equivalent?

The term ‘landscape genomics' is commonly used in the animal field as explained by Farai Muchadeyi. It refers to the animals and their genomes, in the context of geography, rainfall, temperature, humidity, vegetation and pathogens. One example is the adaptation of cattle to their local environment where *Bos taurus* (e.g. the Hereford) thrives in humid temperate European climates but is poorly adapted to tropical and subtropical regions of Africa and elsewhere. Here the *Bos indicus* (e.g. the Brahman) with its longer legs, smooth skin, abundant skin folds and hump, flourishes. Breeds are chosen or adapted to ensure optimal productivity, resistance to pests and diseases and an overall resilience. Is there a place for landscape genomics in human research? It is well known that populations adapt to their environments over several millennia, and that recent migrations, relocations and changes in lifestyle have important implications for health. Examples include the development of vitamin D-deficient rickets in darkly pigmented individuals who move to low UV exposure regions where they do not have access to appropriate nutrition and the dramatic increase in obesity at a population level in urbanizing communities where people have transitioned to a calorie-rich diet and sedentary lifestyle. Research that explores the optimal environment (e.g. diet and behavioural patterns) for people, given their genetic susceptibilities, may lead to stigmatization, despite its intended objectives and would require sensitive public engagement. Poor attempts at translational medicine include misleading advertising in the field of nutrigenomics that has promoted questionable tests and dietary recommendations.

## One health

Bassirou Bonfoh reminded delegates of the concept of One Health that applies to humans, animals and the environment and this found resonance among the group. Local, national and international efforts are essential to integrate the work of multiple disciplines towards optimising universal health.

An obvious focus for the meeting, at the intersection of animal and human health, was zoonotic diseases. There were several presentations on avian and swine flu and pathogenic strains with the ability to cross the species boundaries. However, it became clear that knowledge of prevalence and distribution was scanty, especially in Africa, and the urgency to fund the research leading to the implementation of preventive measures is yet to receive priority in an African context. An inspiring exception is the work of Christian Happi and colleagues in Nigeria on rapid genomics-based detection of outbreaks of infectious diseases such as Ebola and Lassa virus fever.

## Ethics aspects of animal and human genetics

Decision-making and autonomy are different between our animal and human genetics communities in significant ways. Ethics committees ensure that any research on human participants adheres to the four fundamental ethical principles: autonomy, beneficence, non-maleficence and justice. Individuals or their guardians are required to provide informed consent and need to agree that their samples and data may be shared, if this is the intention. In animal research, the emphasis is on respect and balancing general welfare against benefit, while minimizing wastage by choosing appropriate sample sizes. Advanced experiments in or involving animals are also reviewed by institutional animal use and care committees. The objectives can be specifically for commercial gain and in some countries, the decision to share beyond political borders becomes a government decision, where the concept of genomic sovereignty is applied to national fauna and flora. Sharing data from the rich animal genetic diversity can be harnessed to promote genetic improvement. This is in line with the Nagoya Protocol on Access and Benefit Sharing that promotes fair and equitable sharing of the benefits that arise from the use of genetic resources (https://www.cbd.int/).

## Key messages from the meeting

There were four main areas where human and animal health genomics intersected to generate interdisciplinary insights, leading to animated conversations around these subject areas:

### Estimated breeding value in animals, genomic prediction and cross-disorder genetic correlations in humans use very similar analytical approaches and can learn from each other

In animal breeding, the concept of Estimated Breeding Value is assigned to animals and herds for single traits such as milk yield in cattle, to complex health-related and economically valued traits. This requires sophisticated complex genetics analyses across multiple phenotypes. The animal (and plant) genetics and breeding communities have created an entire suite of sophisticated analytical tools to address these issues. In the area of human health, this is echoed by efforts to detect cross-disorder genetic correlations, and pleiotropy. Many of the analytical tools developed in the animal genetics community are now being applied to analyze large human health trait datasets.

### The role of migration in determining patterns of disease susceptibility allele frequencies in humans closely mirrors the concept of Landscape Genetics/Genomics in animal breeding

Migration from one continent or area to another creates different gene–environment interactions relevant to human health. This is still underrecognized by medical professionals, and can lead to suboptimal healthcare. In animal breeding, moving a breed to a new environment similarly creates new challenges, which should first be recognized and then explored, and in some cases harnessed to introduce new measurable traits. Many pertinent examples were discussed in the meeting.

### All of human health and all of animal health are connected, and have been for many thousands of years

The One Health concept as defined by the World Health Organization (https://www.who.int/features/qa/one-health/en/) specifically recognizes that the health of people is connected to the health of animals and the environment. Zoonotic diseases are a pertinent example, and many examples were discussed in the meeting. These re-enforced the notion of a very clear interaction (including various dynamics) between humans and animals, especially in the context of infectious diseases. Sophisticated genomics tools exist to monitor the related dynamics and even anticipate potential movement and adaptation of disease agents (pathogens) between the human, animal and environment interfaces. It became clear during the meeting that we need to think much more deeply about human–animal interactions, and their dynamics, because the disease outbreaks are a symptom of our complex cohabitation and interaction.

### Ethics issues in human and animal health centre around the same normative concept of what constitutes normal, versus artificial or abnormal

Meeting participants grappled with key ethical, moral, social and legal aspects of harnessing genetic diversity for animal and human health. The flexibility of what is permissible in animals does not extend to humans, for example, directed breeding for a desired outcome. Somatic and germline genome editing has already been implemented for economically driven objectives in agriculture and debate is shifting to individual personal choice and the value of every life in medical practice. Human genetics leverages the documentation of genetic diversity, and its link to the phenotype, across the continents and uses this to derive novel biological insights that can be translated into therapies to treat and prevent the symptoms of diseases. Responsible governance of data and biospecimens is a key element to support research and discovery with an emphasis on resource protection to avoid exploitation and potential stigmatization of marginalized communities and leverage of rare breeds, especially in low- to middle-income countries.

## Leveraging infrastructure in LMICs

In regions and countries with resource constraints, infrastructure platforms and data repositories can be shared for research in the health and agricultural sectors. Similarly, analytic and interpretive skills (e.g. biostatistics and bioinformatics) can be developed in synergy to address the most urgent national and regional priorities. Meetings such as the Keystone Symposium can bring key stakeholders together.

## The future—opportunities to explore, characterize and utilize the remarkable animal and human genetic diversity that exists in Africa and other parts of the world

Throughout the meeting, several unifying themes emerged. We measure genetic variation and generate data in the same way in animal and human genetics and have developed similar technological approaches. Our statistical analyses reflect universal principles and applications to population genetics, and we strive to better understand the relationship between genome variation and phenotypes.

In addition to the personal networking and crossover of technological approaches, such as the introduction of multiplexed functional assays for all possible mutations in single human genes (Lea Starita), assessing the genomic context of genetic variants (Nicholas Katsanis) and predicting phenotype from polygenic risk scores, we need to assess the future value of joint human and animal genetics meetings. As we develop an appreciation for the power of genome editing in the animal-breeding world and hotly debate the ethics of this technology in a human context, we need to ensure that the public is well informed and that we as scientists keep the record straight (Alison van Eenennaam).

This meeting aimed to emphasize the practical implementation of genomics approaches, and future joint animal/human genetics meetings could target and explore specific approaches to animal breeding that could be adapted to therapeutic avenues in a clinical setting. In a continent like Africa where scientific resources remain limited, science communication and access to knowledge is essential (Elizabeth Marincola). A follow-on meeting in collaboration with local organizations (i.e. the African Academy of Sciences, AAS; aasciences.ac.ke) could identify key themes to foster a collaboration between the human and animal genetics communities to strengthen funding applications, efficient utilization of resources and even engage with potential private sector collaborators for product development, commercialization and provision of services to drive animal genetic improvement and human health.

Virtual content for the Keystone Symposium: Leveraging Genomic Diversity to Promote Animal and Human Health (available to the broad scientific community without cost).

### Video insight—thought leader summary

https://youtu.be/3fFdv43TXq0

### Virtual access—poster abstracts, scientific talks video recording and ePosters

http://bit.ly/VirtualAccess18S5

### Virtual event—video discussion+Q&A

http://bit.ly/VKSgenomicdiversity

